# The Neonatal Fc Receptor (FcRn) Enhances Human Immunodeficiency Virus Type 1 (HIV-1) Transcytosis across Epithelial Cells

**DOI:** 10.1371/journal.ppat.1003776

**Published:** 2013-11-21

**Authors:** Sandeep Gupta, Johannes S. Gach, Juan C. Becerra, Tran B. Phan, Jeffrey Pudney, Zina Moldoveanu, Sarah B. Joseph, Gary Landucci, Medalyn Jude Supnet, Li-Hua Ping, Davide Corti, Brian Moldt, Zdenek Hel, Antonio Lanzavecchia, Ruth M. Ruprecht, Dennis R. Burton, Jiri Mestecky, Deborah J. Anderson, Donald N. Forthal

**Affiliations:** 1 Division of Infectious Diseases, Department of Medicine, University of California, Irvine School of Medicine, Irvine, California, United States of America; 2 Department of Obstetrics and Gynecology, Boston University School of Medicine, Boston, Massachusetts, United States of America; 3 Department of Microbiology, University of Alabama at Birmingham, Birmingham, Alabama, United States of America; 4 Lineberger Comprehensive Cancer Center, University of North Carolina at Chapel Hill, Chapel Hill, North Carolina, United States of America; 5 Center for AIDS Research, University of North Carolina at Chapel Hill, Chapel Hill, North Carolina, United States of America; 6 Institute for Research in Biomedicine, Bellinzona, Switzerland; 7 Humabs BioMed SA, Bellinzona, Switzerland; 8 Department of Immunology and Microbial Science, International AIDS Vaccine Initiative Neutralizing Antibody Center and Center for HIV/AIDS Vaccine Immunology and Immunogen Discovery, The Scripps Research Institute, La Jolla, California, United States of America; 9 Department of Pathology, University of Alabama at Birmingham, Birmingham, Alabama, United States of America; 10 Institute of Microbiology, Eidgenössische Technische Hochschule (ETH) Zürich, Zürich, Switzerland; 11 Texas Biomedical Research Institute, San Antonio, Texas, United States of America; 12 Ragon Institute of Massachusetts General Hospital, Massachusetts Institute of Technology, and Harvard University, Boston, Massachusetts, United States of America; 13 Institute of Immunology and Microbiology, First School of Medicine, Charles University, Prague, Czech Republic; Miller School of Medicine, United States of America

## Abstract

The mechanisms by which human immunodeficiency virus type 1 (HIV-1) crosses mucosal surfaces to establish infection are unknown. Acidic genital secretions of HIV-1-infected women contain HIV-1 likely coated by antibody. We found that the combination of acidic pH and Env-specific IgG, including that from cervicovaginal and seminal fluids of HIV-1-infected individuals, augmented transcytosis across epithelial cells as much as 20-fold compared with Env-specific IgG at neutral pH or non-specific IgG at either pH. Enhanced transcytosis was observed with clinical HIV-1 isolates, including transmitted/founder strains, and was eliminated in Fc neonatal receptor (FcRn)-knockdown epithelial cells. Non-neutralizing antibodies allowed similar or less transcytosis than neutralizing antibodies. However, the ratio of total:infectious virus was higher for neutralizing antibodies, indicating that they allowed transcytosis while blocking infectivity of transcytosed virus. Immunocytochemistry revealed abundant FcRn expression in columnar epithelia lining the human endocervix and penile urethra. Acidity and Env-specific IgG enhance transcytosis of virus across epithelial cells via FcRn and could facilitate translocation of virus to susceptible target cells following sexual exposure.

## Author Summary

HIV-1 causes a sexually transmitted disease. However, the mechanisms employed by the virus to cross genital tract tissue and establish infection are uncertain. Since cervicovaginal fluid is acidic and HIV-1 in cervicovaginal fluid is likely coated with antibodies, we explored the effect of low pH and HIV-1-specific antibodies on transcytosis, the movement of HIV-1 across tight-junctioned epithelial cells. We found that the combination of HIV-1-specific antibodies and low pH enhanced transcytosis as much as 20-fold. Virus that underwent transcytosis under these conditions was infectious, and infectivity was highly influenced by whether or not the antibody neutralized the virus. We observed enhanced transcytosis using antibody from cervicovaginal and seminal fluids and using transmitted/founder strains of HIV-1. We also found that the enhanced transcytosis was due to the Fc neonatal receptor (FcRn), which binds immune complexes at acidic pH and releases them at neutral pH. Finally, staining of human tissue revealed abundant FcRn expression on columnar epithelial cells of penile urethra and endocervix. Our findings reveal a novel mechanism wherein HIV-1 may facilitate its own transmission by usurping the antibody response directed against itself. These results have important implications for HIV vaccine development and for understanding the earliest events in HIV transmission.

## Introduction

Sexual transmission of HIV-1 requires that virus establish infection across genital tract or intestinal tissue. Sexually transmitted infections, other causes of inflammation, and localized trauma may allow susceptible CD4+ target cells at skin or mucosal surfaces to become directly exposed to secretions from infected sexual partners [1], [2]. However, when skin and mucosa are intact, it remains unclear precisely how HIV-1 gains access to target cells. One possibility is that virus translocates between epithelial cells until susceptible cells are found either in or below the epithelium [3]. Alternatively, Langerhans cells may sample the surface, acquire virus, and move it to areas of abundant target cells [4], [5]. Finally, transcytosis of HIV-1 (i.e., movement through cells) has been studied as a potential mechanism to translocate virus from mucosal surfaces to deeper-lying CD4+ cells [6], [7], [8].

Transcytosis offers an explanation for movement of virus across epithelial cells forming tight junctions, which might normally exclude pathogens from moving beyond the surface. However, *in vitro*, only a very small amount of virus, usually less than 0.3% of a cell-free virus inoculum, finds its way through cells into the medium bathing basolateral surfaces ([9]). Interactions between HIV-1 Env and several host cell surface molecules, including glycolipids, heparan sulfate proteoglycans and gp340, have been proposed to play a role in transcytosis [10], [11], [12], [13], [14].

With the exception of the acute phase prior to development of anti-HIV-1 immune responses, semen, cervicovaginal, and rectal fluids from HIV-1-infected individuals contain antibodies against HIV-1 Env [15], [16], [17]. The concentration of Env-specific IgG present in such secretions varies considerably from person to person and is usually on the order of 100 to 1,000-fold less than concentrations found in plasma [18]. The presence of Env-specific IgG strongly suggests that some proportion of Env molecules on the surface of infectious virions in genital tract secretions is coated with IgG. Since HIV-1 is successfully transmitted sexually, the coating antibody is either of insufficient quantity or quality to neutralize virus infectivity upon contact with an uninfected partner.

Antibody in genital tract secretions of HIV-1-infected individuals could play a role in facilitating the transport of virus across mucosal epithelia. Such a role is made particularly plausible by the reported expression of the Fc neonatal receptor (FcRn) in human genital mucosal tissue [19]. FcRn is a heterodimeric receptor belonging to the MHC class I family of proteins [20], [21]. The expression of FcRn in endothelial cells is thought to be critical for IgG homeostasis in blood [22], and its expression in placental syncytiotrophoblasts is a key factor in transporting maternal IgG to the fetal circulation [23]. A characteristic of FcRn is its ability to bind the Fc region of IgG at acidic pH and to release it at neutral pH [24]. This pH-dependent binding allows the transport of intact IgG or of IgG immune complexes from luminal surfaces bathed in acidic fluids, for example, cervicovaginal secretions, to basolateral surfaces exposed to a neutral intracellular milieu [19].

Cervicovaginal secretions are maintained at acidic pH by acid-producing bacteria that make up part of the normal vaginal microbiota [25]. Although perturbations of normal microbiota, such as occur with bacterial vaginosis, raise the pH, the secretions generally remain in the acidic range [26], [27]. Semen rapidly neutralizes cervicovaginal secretions, but the extent of the pH change is variable. For example, a large amount of ejaculate may raise the pH to the neutral range, whereas a small amount may not [28], [29]. The pH of rectal secretions ranges from about 6.8 to 7.2 [30].

Given that HIV-1 in genital tract secretions may be complexed with IgG antibody, that female genital tract secretions are acidic, and that FcRn has been demonstrated in genital tract tissues, we evaluated the role of pH and antibody on transcytosis of HIV-1 through polarized epithelial cells.

## Results

### Acidic pH and Env-specific IgG enhance transcytosis of HIV-1

**Figure 1:**
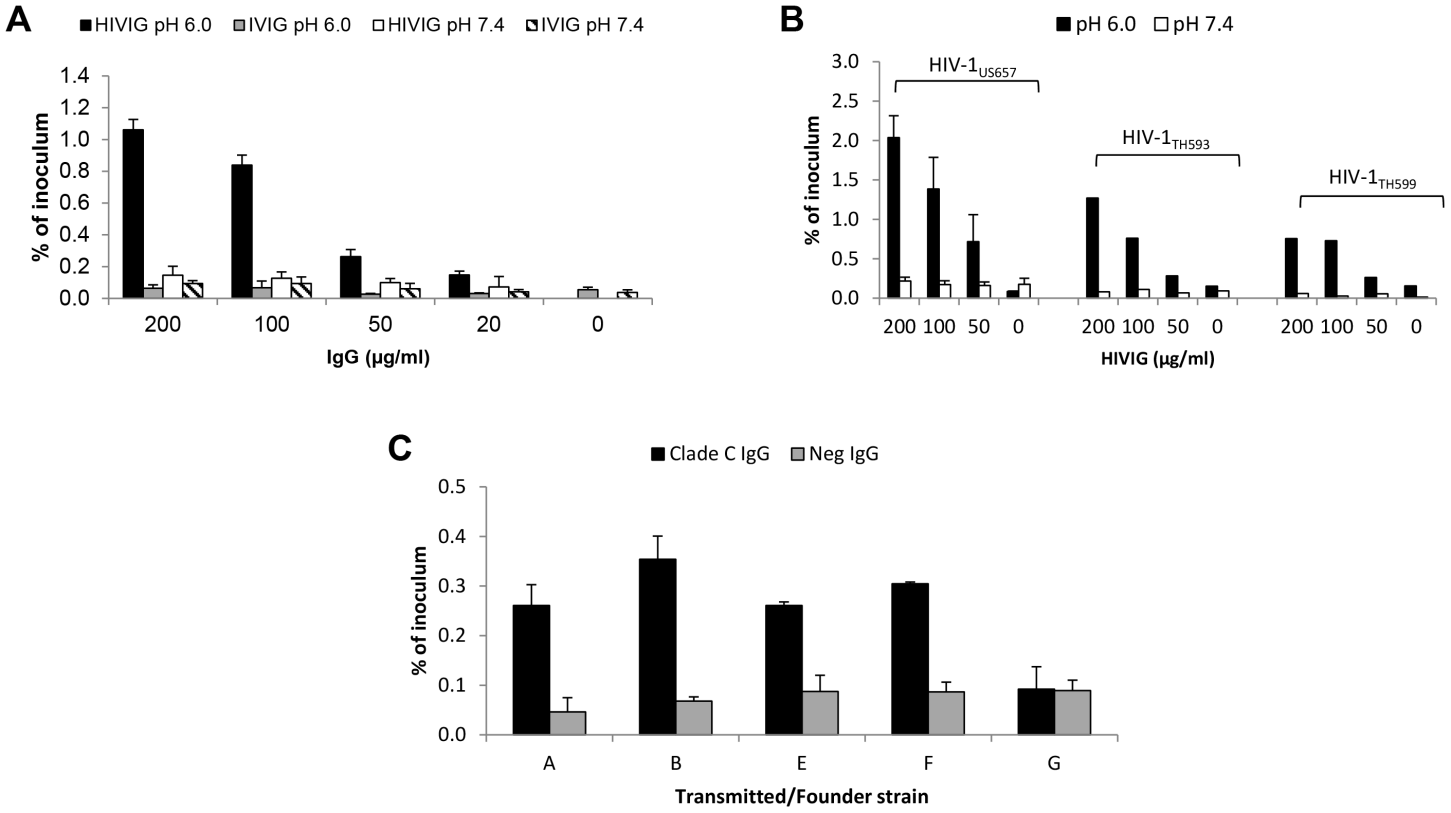
HIV-specific IgG and low pH enhance HIV-1 transcytosis. (**A**) Indicated concentrations of HIV-specific IgG (HIVIG) or control IgG (IVIG) and 2 ng of p24 (average of 3,814,910 RNA copies) of HIV-1_US712_ were added to the apical surface of HEC-1A cells at pH 6.0 or 7.4. Subnatant fluid virus was quantified by RT-PCR and expressed as a percentage of inoculum applied to the apical surface of the HEC-1A cells. Data represent mean+SE of eight independent experiments (some antibody concentrations were tested less frequently); *p* = 3.6×10^−8^ comparing HIVIG at pH 6.0 with IVIG at pH 6.0; *p* = 3.4×10^−6^ comparing HIVIG at pH 6.0 with HIVIG at pH 7.4 (Kruskal-Wallis test). (**B**) Enhanced transcytosis occurs with multiple HIV-1 strains, including clade B R5 (HIV-1_US657_), R5/X4 (HIV-1_HT593_), and X4 (HIV-1_HT599_) strains. Data represent mean+SE of three independent experiments for HIV-1_US657_ (average inoculum = 1,096,491 RNA copies) and one experiment each for HIV-1_HT593_ (inoculum = 2,450,255 RNA copies) and HIV-1_HT599_ (inoculum = 1,477,802 RNA copies). (**C**) IgG enhances transcytosis of clade C transmitted/founder Env-pseudotyped virus. IgG from serum of clade C-infected or uninfected subjects was used at 50 µg/ml. Results are mean+SE of two independent experiments (average inoculum = 1,556,522 RNA copies) at pH 6.0 only; *p* = 0.008 comparing clade C and HIV-Neg IgG (Kruskal-Wallis test).

To investigate the effect of low pH and antibody on HIV-1 transcytosis across epithelial cells forming tight junctions, we exposed the apical surface of HEC-1A cells to HIV-1 at pH 6.0 or 7.4 with or without HIV-1-specific IgG (HIVIG). Virus was quantified in the medium bathing the basolateral cell surface (“subnatant fluid”) by RT-PCR and, although detectable as early as six hours after exposure of virus to the apical cell surface, the quantity was greater at 12 hours (**Figure S1**) [8]. Thus, in subsequent experiments, transcytosis was measured at 12 hours. Using HIV-1_US712_, a clade B R5 clinical isolate, HIVIG enhanced transcytosis in a dose-dependent manner when virus and antibody were exposed to the apical surface at pH 6.0 (**Figure 1A**). There was no increase in transcytosis with HIVIG at pH 7.4 or with HIV-negative IgG (IVIG) at pH 6.0 or 7.4. We found similarly enhanced transcytosis using additional R5 as well as X4 and X4/R5 strains (**Figure 1B**). Importantly, transcytosis of four of five clade C transmitted/founder Env-pseudotyped viruses was enhanced in a pH and antibody-dependent manner (**Figure 1C**). Enhanced transcytosis with HIV-1-specific antibody at low pH also occurred with T84 colon carcinoma cells (**Figure S2**).

Since sexual transmission may occur with small amounts of virus, we investigated if pH- and antibody-dependent enhancement of transcytosis could occur at very low HIV-1 inocula. Using HIVIG or the anti-gp41 monoclonal antibody (mAb) 2F5, we found that transcytosis occurred with virus inocula as low as 2 pg of p24 (about 60,000 RNA copies) with HIVIG and 0.02 pg of p24 (about 500 RNA copies) with 2F5, amounts too small to be detectable in subnatant fluid in the absence of low pH and HIV-1-specific antibody (**Table 1**). These quantities of virus are within the range observed in seminal and cervicovaginal fluids of HIV-infected individuals [31], [32], [33].

**Table 1:** Enhancement of HIV-1 transcytosis occurs with low viral inocula^1^.

Inoculum		Subnatant Fluid (RNA copies)		
p24 (pg)	RNA copies	No antibody	HIVIG	mAb 2F5
2000	64,295,720	250,028	886,060	3,782,575
200	7,949,017	29,223	116,640	362,063
20	652,246	1,983	10,328	34,765
2	62,343	neg	753	4,868
0.2	8,392	neg	neg	658
0.02	523	neg	neg	120

1 Transcytosis carried out at pH 6.0 with 200 µg/ml of HIVIG or 100 µg/ml mAb 2F5; data are representative of three independent experiments.

### Enhanced transcytosis is mediated by FcRn

**Figure 2:**
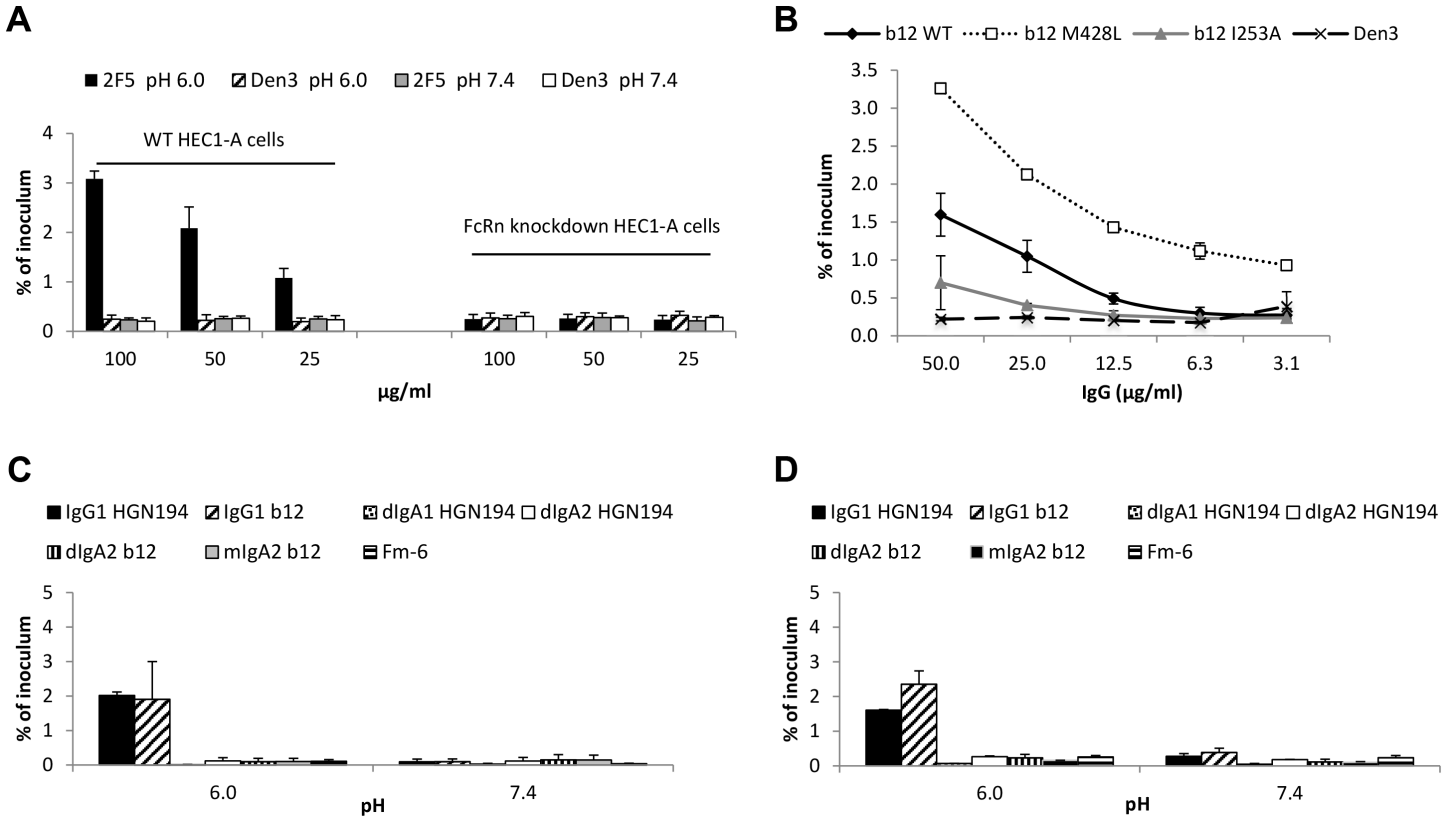
FcRn is responsible for pH/antibody-mediated enhanced transcytosis of HIV-1. (**A**) Knockdown of FcRn eliminates enhanced transcytosis. Transcytosis was performed with the wild-type and FcRn knockdown HEC-1A cells in parallel at pH 6.0 or 7.4 using HIV-1_US657_ (average inoculum = 712,745 RNA copies) and mAb 2F5 or control mAb (Den3). Results represent mean+SE of three independent experiments; *p* = 0.032 comparing wild type and knockdown cells using 2F5 at pH 6.0 (repeated-measures ANCOVA). (**B**) An Fc mutant of mAb b12 that increases FcRn binding (M428L) increases transcytosis of HIV-1_JRFL_ at pH 6.0, whereas a mutant that decreases FcRn binding (I253A) reduces transcytosis. Data represent the mean+SE of two independent experiments. (**C**)(**D**) IgG1, but not IgA1 or IgA2, enhances transcytosis at pH 6.0. mAb IgG1 b12 or its dimeric (dIgA2 b12) and monomeric (mIgA2 b12) class-switched versions and mAb IgG1 HGN194 or class switched variants dIgA1 HGN194 and dIgA2 HGN194 were tested for transcytosis using (**B**) HIV-1_JRFL_ Env-pseudotyped virus (average inoculum = 2,269,529 RNA copies) or (**C**) SHIV_1157ipEL-p_ (average inoculum = 682,724 RNA copies). Fm-6 is an anti-SARS coronavirus IgG1 used as a negative control. All mAbs were tested at 50 µg/ml. Data represent mean+SE of two independent assays for each virus. With either virus, IgG1 versions of the mAbs allow significantly more transcytosis at pH 6.0 than do IgA versions combined (*p* = 0.025 for HIV-1_JRFL_ pseudoviruses and *p* = 0.021 for SHIV_1157ipEL-p_, Kruskal-Wallis test); there is no significant difference between IgG and IgA versions at pH 7.4 (*p*>0.05).

The impact of both antibody and low pH suggested FcRn involvement [34], [35]. We knocked down FcRn in HEC-1A cells, verifying lower expression by flow cytometry and by Western blot (**Figure S3A**). The knock-down HEC-1A cells attained the same level of electrical resistance as did the wild-type cells (data not shown), indicating that FcRn knockdown did not affect the ability to form tight junctions. Unlike with wild-type HEC-1A cells, there was no enhanced transcytosis with FcRn-knockdown HEC-1A cells when either mAb 2F5 (**Figure 2A**) or polyclonal HIVIG (data not shown) were used. We also evaluated Fc mutants of the HIV-1 Env-specific mAb b12. A mutant designed to abrogate FcRn binding (I253A), markedly lowered transcytosis compared with wild-type b12 (**Figure 2B**) [36]. The second mutant (M428L), designed to bind with higher affinity to FcRn, increased transcytosis compared with wild-type b12 (**Figure 2B**) [37]. Binding to HIV-1_JRFL_ gp120 (**Figure S3B**) and neutralization of HIV-1_JRFL_ (**Figure S3C**) were nearly equivalent for the wild-type and Fc mutant versions of b12, indicating that the Fc mutations did not affect Fab-antigen binding. Blockade of FcRn with anti-FcRn antibody and inhibition of endosomal acidification by bafilomycin A1 also substantially reduced or eliminated enhanced transcytosis (**Figure S3D**–**F**), as did competition between the non-HIV-1-specific mAb Den3 and the anti-HIV-1 Env mAb VRC01 (**Figure S3G**). Consistent with other investigations of Fc-FcRn interactions, maximally enhanced transcytosis occurred at pH 5.5–6.0, with some enhanced transcytosis apparent at pH 4.5 and 6.5 (**Figure S3H**) [38], [39]. Since FcRn binds to IgG and not to IgA, we compared two different IgG1 mAbs, b12 and HGN194, with their IgA class-switched versions. Both IgG1 mAbs enhanced transcytosis of HIV-1_JRFL_ pseudoviruses and SHIV_1157ipEL-p_ at pH 6.0, whereas the IgA class-switched versions did not (**Figure 2C and 2D**). In fact, as reported, dimeric IgA1 HGN194 inhibited transcytosis [40]. Thus, enhanced transcytosis at low pH in the presence of specific antibody is mediated by IgG and is dependent on FcRn.

Using 50 µg/ml of VRC01 or Den3, transcytosis of IgG alone increased approximately 3 fold from about 0.4% at pH 7.4 to about 1.3% at pH 6.0 (**Figure S4**). However, the effect of FcRn-mediated transcytosis on IgG alone does not appear as strong as the effect on IgG immune complexes, where, for example, with complexes made with 50 µg/ml of VRC01 or 2F5, there was about an 8-fold increase in transcytosis under conditions allowing FcRn engagement. This difference may be due to the contribution of fluid phase uptake of IgG by the epithelial cells at both pH 6.0 and pH 7.4; IgG thus internalized can engage FcRn in acidic endosomes and be shuttled to the basolateral side of the cells [41]. The internalization of immune complexes, on the other hand, likely depends primarily on FcRn engagement at the surface of the cell at pH 6.0.

### Infectivity of transcytosed virus is affected by antibody function

**Figure 3:**
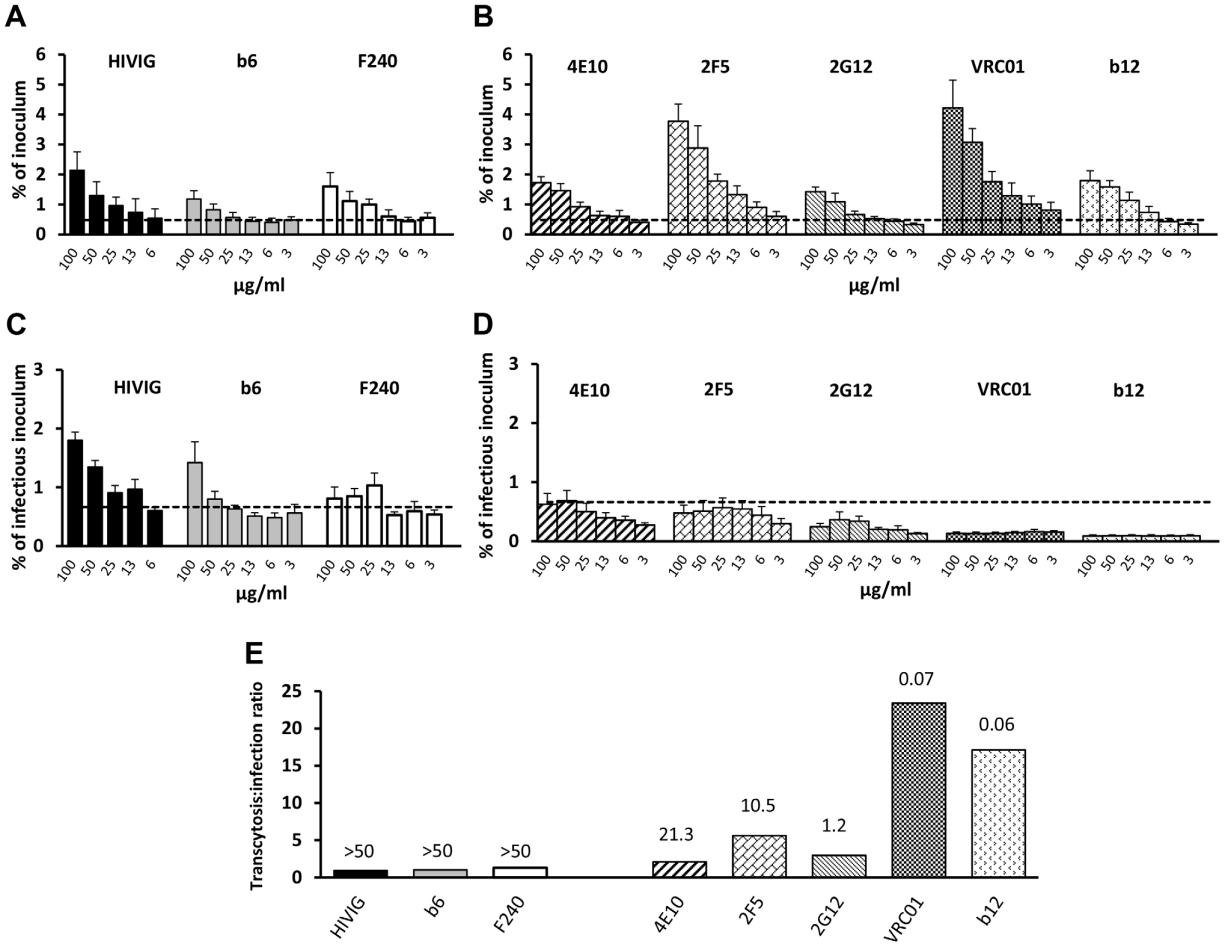
Effect of antibodies on transcytosis and infectivity. Transcytosis (pH 6.0) of HIV-1_JRFL_ by (**A**) poorly neutralizing HIVIG and mAbs b6 and F240 and (**B**) neutralizing mAbs 4E10, 2F5, 2G12, VRC01 and b12. HIV-1_JRFL_ average inoculum = 3,990,745 RNA copies. Dotted lines represent mean+2 SD of % virus transcytosed with Den3 control mAb. There are significant differences between the Den3 control and 2F5 (*p* = 0.0002), b12 (*p* = 0.007), 2G12 (*p* = 0.006), 4E10 (*p* = 0.001), b6 (*p* = 0.008), F240 (*p* = 0.002), and VRC01 (*p* = 0.003) and between IVIG (not shown) and HIVIG (*p* = 0.033) (repeated-measures ANCOVA). (**C**) Infectivity of HIV-1_JRFL_ whose transcytosis was mediated by poorly neutralizing or (**D**) neutralizing antibodies. All antibodies resulted in transcytosed virus that was infectious (>mean+2 SDs of relative light units [RLUs] from uninfected TZM-bl cells [dotted line]); average infectious inoculum = 244,601 RLUs. Compared to Den3 control, less infectious virus was transcytosed with b12 (*p* = 3.4×10^−11^), VRC01 (*p* = 6.9×10^−10^), and 2G12 (*p* = 3.6×10^−4^) (repeated-measures ANCOVA). Compared to Den3, there was more infectious virus with F240 (*p* = 0.036) and with b6 (*p* = 0.068). Compared to IVIG (not shown), HIVIG resulted in more infectious virus (*p* = 0.003). Data in **A**–**D** represent mean+SE of three or four independent experiments. (**E**) Ratio of % transcytosed:% infectious virus from data in (**A**) and (**B**) using 50 µg/ml of antibody. Numbers over bars represent IC_50_ against HIV-1_JRFL_.

We evaluated the ability of HIVIG and a panel of mAbs with variable neutralizing activities to mediate pH-dependent transcytosis with fully infectious HIV-1_JRFL_; 50% inhibitory concentrations (IC_50_s) of the antibodies ranged from 0.06 to >50 µg/ml (**Fig. 3E**). Both poorly neutralizing antibodies (HIVIG and mAbs b6 and F240; **Figure 3A**) and neutralizing mAbs (4E10, 2F5, 2G12, VRC01, and b12; **Figure 3B**) enhanced transcytosis at pH 6.0. At 50 µg/ml, transcytosis correlated directly with mAb binding to HIV-1_JRFL_ (Spearman *rho* = 0.75; *p* = 0.052) and inversely with the IC_50_ of the mAbs (Spearman *rho* = −0.71; *p* = 0.050) (**Figure S5**). At pH 6.0, all Env-specific mAbs and HIVIG mediated transcytosis of virus that infected TZM-bl cells (**Figure 3C and D**). However, there was a strong correlation between the amount of transcytosed infectious virus and the neutralizing activity (IC_50_) of the antibody that mediated the transcytosis (Spearman's *rho* = 0.86; *p* = 0.001). Virus whose transcytosis was mediated by poorly neutralizing antibodies HIVIG, F240 and b6, at least at concentrations of 100 and 50 µg/ml, was more infectious than virus which crossed the epithelial cells in a non-FcRn-dependent manner (i.e., in the presence of Den3 control mAb) (**Fig. 3C**). Conversely, transcytosis mediated by antibodies with the lowest IC_50_s, such as VRC01 and b12, resulted in less infectious virus than was observed with the Den3 control antibody (**Figure 3D**). Thus, strong binding activity results in more FcRn-dependent transcytosis, whereas strong neutralizing activity renders the transcytosed virus less infectious. This point is further illustrated by the ratio of percent-transcytosed:percent-infectious virus (**Figure 3E**). For example, for every infectious unit, about 30 times more virus is transcytosed with VRC01 than with HIVIG (**Figure 3E**). Note that independently of transcytosis, HIV-1_JRLF_ infectivity on TZM-bl cells increased about 3.5-fold after incubation of virus for 12 hours at pH 6.0 compared with pH 7.4; however, IC_50s_ were very similar (<15% difference) at the two pH values (pH comparisons done for 2F5 and VRC01 only; data not shown). Virus infectivity was essentially abrogated after a 12-hour incubation at pH 4.0 (data not shown).

### IgG from genital tract secretions enhances transcytosis at pH 6.0

**Figure 4:**
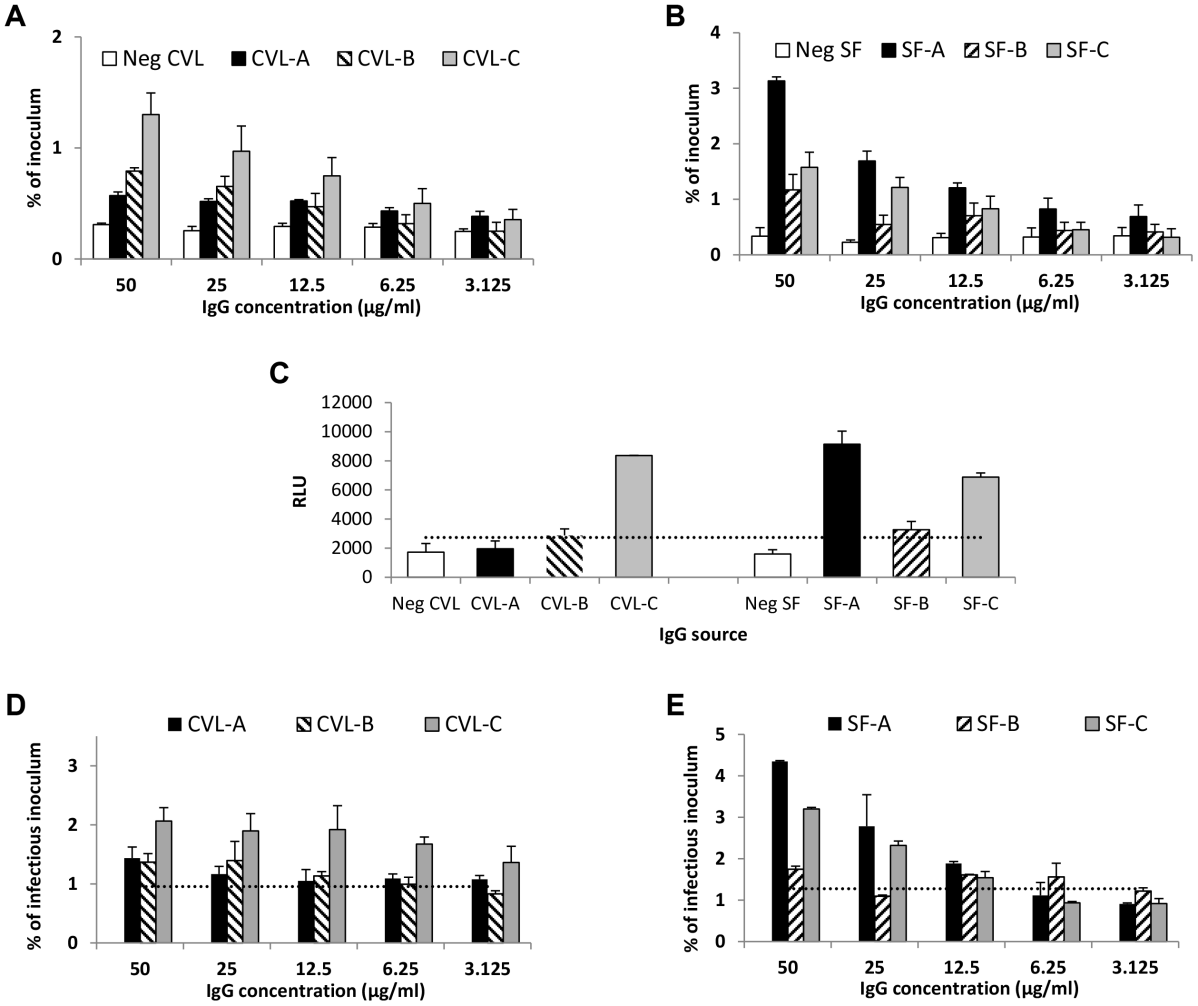
IgG purified from genital tract secretions enhances transcytosis at pH 6.0. (**A**) Cervicovaginal (CVL) fluid IgG from three HIV-infected women (CVL-A, CVL-B, and CVL-C) mediates enhanced transcytosis of HIV-1_US657_ (average inoculum = 2,479,412 RNA copies) at pH 6.0. (**B**) Similarly, IgG purified from seminal fluid of three HIV-infected men (SF-A, SF-B, and SF-C) enhances transcytosis of HIV-1_US657_ (average inoculum = 622,642 RNA copies) at pH 6.0. (**C**) Infectious HIV-1 capture by antibody in CVL and seminal fluids. Virus was captured by the indicated IgG (5 µg/ml) and added to TZM-bl cells; infection was then quantified by RLUs. Infectious virus capture correlates with transcytosis at pH 6.0 (Spearman's *rho* = 0.94; *p* = 0.005). Shown is the mean+SE of duplicate samples. The dotted line represents a negative cutoff based on the mean+2 SD of IgG from cervicovaginal or seminal fluid of HIV-uninfected subjects. (**D**) (**E**) Both cervicovaginal fluid IgG (**D**) and seminal fluid IgG (**E**) mediate transcytosis of infectious virus. Average infectious inoculum = 1,160,894 and 1,913,288 relative light units [RLUs] for cervicovaginal and seminal fluid, respectively. The dotted lines represent negative cutoffs based on the mean+2 SD of IgG from cervicovaginal and seminal fluid of the HIV-uninfected subjects. Data shown (**A**, **B**, **D**, and **E**) are mean+SE of two independent assays.

We next determined whether IgG purified from cervicovaginal fluid and from seminal fluid could enhance transcytosis at low pH. Using IgG from cervicovaginal fluid of three HIV-infected women and from seminal fluid of three infected men, enhanced transcytosis occurred at IgG concentrations well within their expected range in genital tract secretions (**Figure 4A and 4B**) [18]. The ability of genital tract IgG to mediate transcytosis correlated strongly with infectious virus capture activity by the IgG (Spearman's *rho* = 0.94, *p* = 0.005; **Figure 4C**) and less so with binding to monomeric Env glycoprotein from the same virus strain (HIV-1_US657_) (*rho* = 0.65, *p* = 0.16; **Figure S6**). None of the genital tract IgGs were able to neutralize HIV_US657_, the clinical R5 strain used in these experiments, at IgG concentrations as high as 50 µg/ml (not shown). Consistent with HIVIG and the non-neutralizing mAbs, higher concentrations of genital tract IgGs generally resulted in greater infectivity of the transcytosed virus (**Figure 4D and 4E**).

### FcRn is expressed in genital tract tissues

**Figure 5:**
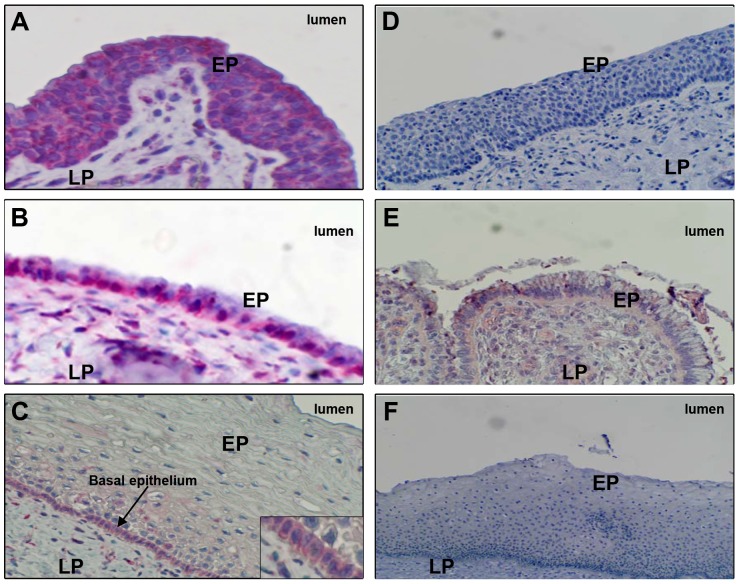
FcRn expression in human genital tissues, detected by immunohistochemistry. FcRn expression (red stain) in columnar epithelia lining (**A**) the penile urethra (representative of 16 donors), (**B**) endocervix (representative of five donors), or (**C**) vagina (representative of 12 donors). Tissues were also stained with negative control (non-specific) IgG and processed through the same immunohistochemistry procedure: (**D**) penile urethra, (**E**) endocervix, and (**F**) vaginal epithelia. Epithelia (EP) and lamina propria (LP) are labeled. Staining was performed on paraffin-embedded specimens.

FcRn expression was previously reported in human uterine and vaginal epithelial cells [19]. Using immunohistochemistry to survey FcRn protein expression at various sites in the human genital tract, we detected abundant FcRn expression in columnar epithelial cells lining the human penile urethra (**Figure 5A and 5D** **; Figure S7A** and **S7D**) and endocervix (**Figure 5B and 5E** **; Figure S7B** and **S7E**). In contrast, little to no FcRn protein was observed in vaginal/ectocervical squamous epithelia, and expression occurred only in the basal epithelial layer (**Figure 5C and 5F** **; Figure S7C**). A similar staining pattern was observed in foreskin tissue (data not shown).

## Discussion

Female genital tract secretions are often acidic, and the secretions of HIV-infected individuals have antibody capable of coating virus contained in those secretions. These facts led us to explore the role of antibody and low pH on transcytosis of HIV-1 across epithelial cells. Our primary finding is that at acidic pH, IgG enhances transcytosis of HIV-1 clinical isolates, including transmitted/founder Env-pseudotyped strains. Moreover, antibody from both cervicovaginal and seminal fluid mediates enhanced transcytosis at low pH. The enhanced transcytosis is abrogated by blocking or knocking down FcRn, which is known to bind IgG and immune complexes at low pH and release them at neutral pH [24], [42]. We also establish that virus translocated across epithelial cells after incubation with antibody at low pH remains infectious. Although neutralizing antibodies generally promote more transcytosis, the transcytosed virus is relatively less infectious than virus whose transcytosis is mediated by non-neutralizing antibodies. Finally, we demonstrate abundant FcRn protein expression in columnar epithelial cells of the human endocervix and penile urethra, suggesting that these sites could play a major role in FcRn-mediated immune complex transcytosis.

Our results indicate that FcRn may be responsible for shuttling IgG-bound HIV-1 across epithelial cells in the genital tract. This is consistent with other studies that have highlighted a role for FcRn in immune complex shuttling across tissues [34], [35], [43]. Mice expressing human FcRn in intestinal epithelial cells were able to deliver IgG to the luminal intestinal surface, which could then bind to its cognate antigen and return the immune complex back to the lamina propria for presentation by dendritic cells to CD4+ T cells [34]. In addition, cytomegalovirus (CMV) applied to human placental explants from women with high anti-CMV neutralizing antibody activity was rapidly transcytosed across syncytiotrophoblasts and captured by villus macrophages [35]. Under these conditions, the virus did not replicate. However, in explants from CMV-seropositive women with low or undetectable neutralizing antibodies, virus replication readily occurred in cytotrophoblasts underlying an intact, uninfected syncytiotrophoblast layer. Thus, it appeared that neutralizing antibody inhibited infection after allowing virus to cross the syncytiotrophoblast layer. On the other hand, non-neutralizing antibody allowed or even promoted infection. Syncytiotrophoblasts express high levels of FcRn, and when FcRn on explants was blocked, IgG-virion complexes were not transported across the surface [35]. Just as we found with HIV-1, FcRn-mediated transcytosis of CMV occurred with both neutralizing and poorly neutralizing antibody, but transcytosed virus remained infectious only when complexed with poorly neutralizing antibody. Finally, immunohistochemical staining of placentas from *in utero* infections were consistent with this model of FcRn-mediated transcytosis [35].

To our knowledge, ours is the first study to investigate transcytosis using virus coated with HIV-specific antibody in an acidic environment that mimics that of the female genital tract. Our *in vitro* observations are applicable to male-to-female transmission via vaginal intercourse, where enhanced transcytosis could facilitate infection. In this regard, Li et al. reported FcRn expression and bidirectional IgG transport in a human vaginal tissue model [19]. Although we did not detect FcRn in the apical layers of vaginal epithelium, we did detect abundant FcRn expression in columnar endocervical epithelial cells. These cells may be exposed to acidic vaginal secretions where they occur at the cervical os. Furthermore, cervical ectopy, a common condition characterized by the extension of endocervical columnar epithelium into the ectocervix and upper vagina, has been implicated as a risk factor for HIV-1 infection [44], [45]. Prevalent in reproductive-age women, these cervical lesions are exposed to vaginal pH conditions and could provide portals for FcRn-mediated male-to-female HIV-1 transmission [46]. FcRn was also found, though not consistently, in basal epithelial cells of the vagina. These cells lie deep in the epithelium and are unlikely to come in contact with acidic secretions and HIV-1 immune complexes unless there were trauma or substantial thinning of the overlying squamous epithelium. It is important to note that seminal fluid can rapidly raise the pH of cervicovaginal secretions to levels which would not support immune complex-FcRn binding. However, the pH of cervicovaginal fluid following ejaculation is dependent on the quantity of the ejaculate and may stay within an acidic range [29]. Furthermore, HIV is present in preejaculate secretions and could be introduced into the female genital tract prior to ejaculation [47].

With respect to female-to-male transmission, the penis comes in contact with vaginal secretions that would remain at acidic pH at least until ejaculation, allowing time for exposure of penile tissues, including the foreskin and urethra, to IgG-coated virus at low pH [28], [29]. Our demonstration of abundant FcRn on human penile urethral epithelium supports a model where exposure to antibody-bound HIV-1 might lead to enhanced female-to-male transmission. It should be noted that the pH of vaginal secretions is typically about 4, which is below the pH required for Fc-FcRn binding [48]. However, there is substantial variability in normal vaginal pH [26], [48], and we did begin to observe enhanced transcytosis at pH 4.5 (**Figure S3H**). Furthermore, it is possible that there is some buffering effect of foreskin and urethral secretions. The foreskin, whose presence increases HIV infection rate, could trap secretions containing HIV-1 immune complexes and thereby allow greater urethral exposure to infected material within the pH range of Fc-FcRn binding [49]. Additionally, bacterial vaginosis, a condition associated with an increased risk of female-to-male (as well as male-female) HIV transmission, results in vaginal secretions ideal for Fc-FcRn binding [26], [27], [50]. Exposure of penile tissues to the pH range of Fc-FcRn binding may also occur after ejaculation, since complete neutralization of vaginal acidity may not occur immediately or at all [29]. It is also possible, though less likely, that FcRn mediates HIV transmission via the penis during insertive anal intercourse, where the penis may come into contact with slightly acidic rectal secretions [30].

The finding that IgG from cervicovaginal and seminal fluids obtained from HIV-infected individuals mediate enhanced transcytosis of infectious virus further suggests the biological relevance of our results. Cervicovaginal and seminal fluids are reported to contain an average of ∼3 µg/ml and up to ∼15 µg/ml of Env-specific IgG [18]. Four of the six samples we evaluated bound to infectious HIV-1 at 5 µg/ml. Moreover, all of our samples mediated transcytosis at ≤12.5 µg/ml of total IgG, well below total IgG concentrations found in genital secretions of HIV-infected men and women [18]. Even during acute HIV infection, when the risk of transmission to an uninfected partner is highest, 23 of 23 subjects (100%) were reported to have anti-gp41 IgG antibodies and 40% had anti-gp120 IgG antibodies in cervicovaginal and seminal fluids [17]. Anti-gp41 IgG levels were on average 11-fold higher than gp41-specific IgA levels; anti-gp41 IgM was found less frequently and in lower quantity. Thus, HIV-1 immune complexes are likely to occur in mucosal secretions, are likely to contain predominantly IgG, and under acidic conditions, would be subject to FcRn-mediated transcytosis in an exposed host.

The relevance of our findings is also supported by our demonstration that transcytosis of transmitted/founder strains of HIV-1 Env pseudotyped virus is enhanced by antibody. We are currently evaluating whether transmitted/founder strains, in comparison with chronic strains, are preferentially transcytosed, which would be consistent with a report showing a higher sensitivity of clade B transmitted/founder strains to anti-Env antibody binding [51].

Our findings represent a new model of antibody-dependent enhancement (ADE) of HIV-1 infection. Previous studies have demonstrated ADE *in vitro* due to FcγR- or complement-mediated mechanisms or to modulation of the interaction of gp120 with CCR5 [52], [53], [54]. Here we demonstrate that enhancement *in vitro* occurs at the level of transcytosis across epithelial cells and involves FcRn. *In vivo*, Ig isotype, as well as neutralizing activity, are likely to play a determining role in whether an antibody might protect from or enhance infection. As demonstrated recently, intrarectally applied dIgA1 HGN194 mAb, but less so the IgG1 version, prevented SHIV infection following intrarectal challenge [40]. *In vitro*, the dIgA1 inhibited transcytosis, whereas we now show that the IgG1 version enhances transcytosis at pH 6.0. Another study showed that, compared to irrelevant- and no-antibody controls, there was an increase in the number of transmitted/founder SHIV variants when vaginal challenge followed systemic or local infusion of a non-neutralizing IgG1 mAb [55]. Clearly, other studies have found that IgG with neutralizing activity can prevent lentivirus infection after vaginal challenge [56], [57]. Thus, whereas a strong vaccine-induced neutralizing IgG response may protect, non-neutralizing IgG or waning titers of neutralizing IgG present in an acidic lumen might enhance transcytosis across mucosal barriers while allowing infection of susceptible target cells. However, whether an antibody protects, enhances or has no effect is likely to depend on the potency and breadth of antiviral activity, the viral strain, the inflammatory state of the exposed individual, and genetic factors—such as FcγR polymorphisms—that might influence antibody function [58]. Finally, if FcRn-mediated transcytosis applies *in vivo*, our results would strengthen the argument for a mucosal IgA response to vaccination—though not at the exclusion of a strong IgG neutralizing or other anti-viral response—since IgA can inhibit transcytosis, would not engage FcRn, and mediates only uni-directional translocation of immune complexes from the subepithelial space into external secretions [40], [59].

Some studies have reported that anti-HIV-1 Env IgG antibodies can inhibit transcytosis [9], [60], [61]. One of these studies found that polyclonal anti-HIV Env IgG inhibited transcytosis of cell-free virus on HEC-1 cells, whereas none of 13 mAbs did; in fact, some of the mAbs might have increased transcytosis, although by no more than about 50% [61]. To our knowledge, none of these studies was carried out under the acidic conditions that characterize female genital tract secretions.

Our results suggest that FcRn might facilitate infection in hosts without pre-existing antibody or with a non-neutralizing IgG response to prior infection (which would result in secondary infection) or to vaccination. However, FcRn could also play a beneficial role in preventing infection after exposure. FcRn mediates the bidirectional transcytosis of IgG, and in immunized individuals, could provide a conduit for antibodies to neutralize virus as shown for herpes simplex virus type 2 [19]. In addition, IgG immune complexes can prime CD4+ and CD8+ T cells in an FcRn-dependent manner, and FcRn targeting may be a useful mucosal immunization strategy [62], [63], [64].

In summary, we have demonstrated that FcRn mediates enhanced transcytosis of HIV-1 in the presence of low pH and HIV-1-specific antibody. We have also shown that FcRn is present on epithelial cells in areas of the genital tract that are potentially exposed to HIV-1 during sexual intercourse. Our findings point toward a novel mechanism by which the sexual transmission of HIV-1 may be facilitated.

## Methods

### Ethics statement

This research was approved by the Institutional Review Boards at the University of California, Irvine, Boston University, and the University of Alabama, Birmingham. Subjects from whom specimens were collected for study purposes provided written informed consent.

### Cell lines, cell culture media, and viruses

Human Endometrial Carcinoma (HEC-1A) cells (ATCC) were propagated in Modified McCoy's 5a Medium, and Human Colon Carcinoma (T84) cells (ATCC) in Dulbecco's modified Eagle's medium; media were supplemented with 2.5 mM L-glutamine (Gibco, Invitrogen Technologies), 1% Penstrep (Cellgro Mediatech Inc.) and 10% FBS (Atlas Biologicals) and maintained at 37°C with 5% CO2. TZM-bl cells (NIH AIDS Reagent Program) for infectivity assays were propagated in RPMI 1640 supplemented with L-glutamine, Penstrep and 10% FBS as above. Five primary clinical HIV-1 strains, HIV-1_US657_, HIV-1_US712_, HIV-1_JRFL_, HIV-1_HT593_, and HIV-1_HT599_ were obtained from the NIH AIDS Reagent Program. SHIV_1157ipEL-p_, provided by Ruth Ruprecht, was grown in rhesus peripheral blood mononuclear cells [65].

### Antibodies

HIVIG (IgG derived from pooled plasma of HIV-infected individuals) and IgG1 monoclonal antibodies (mAbs) 2F5, 4E10, 2G12, F240, b6, and VRC01 were obtained from the NIH AIDS Reagents Program. IVIG (Gamunex, Taleris Biotherapeutics) was commercially acquired. mAb b12 and control mAb Den3 were provided by Dennis Burton and Brian Moldt, and control mAb Fm-6 was a gift of Wayne Marasco (Dana-Farber Cancer Institute); b12 and the control mAbs are IgG1. Generation and purification of dimeric and monomeric IgA2 versions of b12 (dIgA2 b12 and mIgA2 b12) are described elsewhere [66]. Briefly, the IgG constant region in pDR.12 (IgG b12) was replaced with the constant region of IgA2. IgA2 b12 was expressed in CHO-K1 cells with human J chain and purified by Protein L affinity matrix (Pierce). mIgA b12 and dIgA b12 were isolated by size exclusion chromatography. IgG1 HGN194 (a human mAb against HIV-1 Env V3), dIgA1 HGN194, and dIgA2HGN194 were provided by Davide Corti and Antonio Lanzavecchia [67]. HGN194 variants were constructed as follows: human J chain precursor (accession number NP_653247), IgA1 (allele IGHA1*01, accession number J00220) and IgA2 (allele IGHA2*01, accession number J00221) constant region nucleotide sequences were codon optimized and synthesized by Genscript. Constant regions were cloned into a mammalian expression vector used for subcloning of the HGN194 VH region. The HGN194 VH and VL chain were codon optimized and synthesized by Genscript and cloned into an IgG1 and Ig-lambda expression vector. MAbs HGN194 dIgA1, dIgA2, and IgG1 were produced by transient transfection of 293 freestyle cells with polyethylenimine and expression plasmids encoding corresponding heavy and light chains (in the case of dIgA1 and dIgA2, the J chain expression plasmid was included). Supernatant fluid from transfected cells was collected after 7–10 days of culture. HGN194 dIgA1, dIgA2, and IgG1 were affinity purified by Peptide M (dIgA1 and dIgA2) or Protein A (IgG1) chromatography. Purified Abs were quantified by ELISA using dIgA1 and dIgA2 or IgG1-specific Abs (Southern Biotech). Purity and polymeric state of dIgA1 and dIgA2 were confirmed by native-PAGE analysis and gel filtration chromatography. The presence of dIgA1 and dIgA2 associated J-chain was confirmed by Western blot from native and SDS-PAGE gels. Sera from 20 Zambian clade C-infected subjects (obtained from Zdenek Hel, University of Alabama, Birmingham) were pooled for IgG isolation using the Pierce Melon Gel IgG Spin Purification Kit (Thermo Scientific) according to the manufacturer's instructions. Env-specific IgG, determined as for CVL and seminal fluid (see below), was 0.98% of total IgG. Sera from five uninfected individuals were pooled and processed for IgG isolation in the same manner.

Fc mutants designed to enhance (M428L) or reduce (I253A) mAb b12 binding to FcRn were constructed as follows: briefly, the b12 variable regions were PCR-amplified from pDR12 and cloned into the pγ1HC and pκLC vectors [68], [69]. Amino acid substitutions were introduced by QuikChange site-directed mutagenesis (Stratagene, La Jolla, CA). Constructs were verified by sequence analysis before transiently expressed in FreeStyle 293 cells (Invitrogen, Carlsbad, CA) and purified by protein A affinity chromatography (GE Healthcare, United Kingdom).

### Virus neutralization assay

Antibodies were tested for neutralizing activity against indicated HIV-1 strains using TZM-bl cells.

### Virus capture assay

Half-area 96-well plates (Corning) were coated with 5 µg/ml (250 ng/well) of goat anti-human Fc antibody and incubated over night at 4°C. Plates were then washed with PBS and blocked with 4% non-fat dry milk for 1 hour at room temperature (RT). After washing, capture antibodies were added at 5 µg/ml (250 ng/well), and plates were incubated an additional hour at RT. Next, virus was added to washed plates (20 ng p24/well) and incubated for 3 hours at 37°C. Unbound virus was removed by washing with PBS. Subsequently, 1×10^4^ TZM-bl cells/well were added in the presence of 10 µg/ml DEAE dextran and incubated for 48 hours at 37°C. Cells were then washed, lysed, and developed with luciferase assay reagent according to the manufacturer's instructions (Promega). Luminescence (relative light units) was measured using a Synergy 2 microplate luminometer (BioTek).

### Antibody binding assays

We measured binding of antibodies either to virus directly coated on ELISA-plate wells or to solubilized Env. For the direct virus binding assay, plates were coated with HIV-1_JR-FL_ (20 ng p24/well) for 2 hours at 37°C, washed with PBS and blocked with 4% non-fat dry milk in PBS. After 1 hour at 37°C, plates were washed, antibodies were added in serial dilutions and incubated for 1 hour at 37°C. Detector antibody (horse radish peroxidase-labeled goat anti-human Fc) was added to the washed plate and incubated for 45 min at 37°C. Finally, plates were washed, developed (TMB solution, Life Technologies), and read at 450 nm using a plate reader (BioTek).

The soluble Env binding assay was performed as previously described with some modifications [70]. Briefly, wells were coated with 250 ng of a gp120 Env specific anti-C5-antibody (D7324 [Aalto Bioscience]), washed and blocked with 4% non-fat dry milk. Serial dilutions of detergent-solubilized HIV-1_JR-FL_ (starting at 150 ng p24) was added and incubated for 2 hours at 37°C. Plates were then incubated with a constant concentration of antibodies (1 µg/mL) for 1 hour at 37°C followed by detection and development steps as described above.

### Clade C transmitted/founder Env pseudotyped virus

Five R5 clade C transmitted/founder Env pseudotyped strains were constructed as described [71], [72]. Briefly, rev-vpu-env cassettes from the transmitted founder strains were cloned into pcDNA 3.1D/V5-HIS TOPO® expression vector. The pseudotyped viruses were then produced by co-transfecting 293T cells with pcDNA 3.1(rev-vpu-env), pNL4-3.lucR-E-, and fugene 6 (Roche).

### Cervicovaginal and seminal fluid IgG

Cervicovaginal lavage (CVL) and seminal fluid were collected from HIV-1-infected patients and healthy volunteers at the University of Alabama, Birmingham. All subjects gave written consent in accordance with an IRB-approved protocol. CVL was collected from one 34 year-old uninfected women and from three infected women (age 29 to 46 years) with CD4+ lymphocyte counts of 458/mm^3^, 181/mm^3^ and 498/mm^3^ and plasma viral loads of 14100 copies/ml, 824 copies/ml and 88 copies/ml, respectively. Viral loads were not measured in the CVL fluid specimens. Two of the women (with the lower plasma viral loads) were receiving anti-retroviral therapy. Briefly, CVL fluid was obtained by flushing the cervix and vagina with 5 ml sterile saline, and the wash was collected into tubes with protease inhibitors ([73]). Seminal fluid was obtained from two uninfected men (ages 25 and 40 years), and from three infected men (age 43 to 53 years) by masturbation (58). CD4 counts in the infected men were 404/mm^3^, 336/mm^3^ and 407/mm^3^ and plasma viral loads were <100 copies/ml, 8092 copies/ml and 6750 copies/ml, respectively; only one of these subjects (with viral load of 11 copies/ml) was receiving anti-retroviral therapy. Seminal fluid was assayed for HIV-1 RNA by PCR, but none was detected. The cervicovaginal and seminal fluids were centrifuged and supernatant fluids aliquoted and frozen at −80°C until assayed. Total IgG was determined by ELISA [74]. IgG isolation was accomplished by incubating samples with Protein G-Sepharose (GE Healthcare Bio-Sciences Corp.) followed by elution of bound IgG according to manufacturer's instructions. The IgG preparations were concentrated and dialyzed against DPBS using Amicon Centrifugal Filter Units (Millipore Corp.). The IgG preparations from the two uninfected men were pooled to obtain sufficient quantity for experiments; all other IgG preparations were tested individually.

Env-specific IgG binding levels in seminal and CVL fluids were quantified by ELISA. Detergent-solubilized Env from HIV-1_US657_ was captured by a polyclonal sheep anti-gp120 antibody (D7324, Aalto Bio Reagents Ltd). Sample IgG and an anti-gp120 mAb standard (b6) were serially diluted, added to wells, washed, and detected by anti-human IgG (gamma)-HRP (Sigma-Aldrich, A6029) antibody. Plates were subsequently developed, stopped, and read at OD_450_ nm. The concentrations of Env-specific IgG in the seminal and CVL IgG samples were calculated using the mAb b6 standard and are reported as a percent of total IgG in each sample.

Anti-Env IgG ranged from 0.9 to 2.6% of total IgG in the seminal fluid specimens and from 0.1 to 0.6% of total IgG in the CVL fluids specimens (**Figure S6**).

### Dual chamber transcytosis assay

Transcytosis assays were conducted using reproductive tract-derived (human endometrial carcinoma [HEC-1A]) or intestinal tract-derived (human colonic carcinoma [T84]) cells. HEC1-1A or T84 cell monolayers were created on 0.4 µm polyethylene terephthalate membrane hanging transwell inserts (Millipore). Cell viability was >95% at the time of plating. Electrical resistance across the membrane, which ranged from 400–450 mOhms/cm^2^ at the start of the transcytosis assay, confirmed monolayer integrity. Resistance was re-measured after the transcytosis assay in more than 50% of wells and ranged from 450–480 mOhms/cm^2^. HIV-1 alone or with antibody was added to monolayers in media buffered to pH 6.0 or 7.4. After 12 hours, fluid in the lower chamber (“subnatant fluid”), maintained at pH 7.4, was collected and used to measure viral RNA copy number and infectivity. In the absence of cell monolayers, about 69% of the virus inoculum was present in the lower chamber of the wells after 12 hours.

### Viral RNA extraction and quantitative RT-PCR

Viral RNA was extracted from cell-free subnatant fluid using PureLink Viral RNA Mini Kits (Invitrogen) or NucleoSpin RNA Virus extraction kits (Macherey Nagel Inc.), according to the manufacturers' instructions. Quantitative one-step real-time RT-PCR of extracted HIV-1 viral RNA was done using Quantitect SYBR Green RT-PCR kits (Qiagen GmbH) and that of SHIV_1157ipEL-p_ with Rotor Gene Probe RT-PCR kits according to the manufacturers' instructions. HIV-1gag primers: SK462 d(AGTTGGAGGA-CATCAAGCAGCCATGCAAAT) and SK431 d(TGCTATGTCAGTTCCCCTTGGTTCTCT) (AnaSpec Inc.). SIV-1 *gag* primers: d(GGG AGA TGG GCG TGA GAA A) and d(CGT TGG GTC GTA GCC TAA TTT T). SIV-1 *gag* probe: d(TCA TCT GCT TTC TTC CCT GAC AAG ACG GA) (Integrated DNA Technologies, Inc.).

### Infectivity assays

150 µl of subnatant fluid was used to infect 1×10^4^ TZM-bl cells. TZM-bl cells were lysed 2 days post-infection with 1X Cell Culture Lysis Reagent (Promega Corp.), and luciferase activity was determined by chemiluminescence using Luciferase Substrate (Promega Corp.).

### FcRn knockdown of HEC-1A cells

HEC-1A cells were transduced with FcRn shRNA Lentiviral Particles (Santa Cruz Biotechnology Inc.) following manufacturer's protocol. Cells were selected in medium containing 5 µg/ml Puromycin dihydrochloride (Sigma-Aldrich Inc.), and FcRn expression was verified by flow cytometry using rabbit polyclonal anti-FcRn antibody (Santa Cruz Biotechnology Inc.), normal rabbit IgG (negative control) and FITC-goat anti-rabbit IgG F(ab′)2 secondary antibody (Jackson ImmunoResearch Laboratories Inc.) (**Figure S3A**). Cytofix/Cytoperm Plus Kits (BD Biosciences) were used to fix, permeabilize and stain cells. Knockdown of FcRn was also confirmed by western blot using rabbit anti-FcRn antibody (Novus Biologicals) (**Figure S3A**). Wild-type and knockdown cells had similar viability. Neither wild-type nor knockdown HEC-1A cells stained for FcγRIIa or FcγRIIIa (not shown).

### FcRn blocking and inhibition of endosomal acidification

HEC-1A cells were incubated with 50 µg/ml rabbit polyclonal anti-FcRn IgG (H-274; Santa Cruz Biotechnology Inc.) or normal rabbit polyclonal IgG for 1 hour at pH 7.4 before exposing the apical surface to HIV-1_US712_ and HIVIG, b12, IVIG or Synagis. Similarly, HEC-1A cells were incubated with 0.1 µM bafilomycin A1 (Santa Cruz Biotechnology Inc.) for 1 hour prior to HIV-1 and antibody exposure. Transcytosis was then carried out as above.

### Detection of FcRn protein expression in human genital tissues by immunohistochemistry

Cervical tissue, which included portions of endocervix and upper vagina (ectocervix), was obtained from 10 women aged 31–50 undergoing hysterectomy for nonmalignant conditions. Vaginal tissue was also obtained from women undergoing vaginal repair (n = 6, aged 44–78 years). Penile tissue, including urethra (n = 16) and foreskin (n = 2), was harvested at autopsy from 16 men aged 34–73 with no history of hormonal or immunosuppressive medications. Tissues were processed within 60 minutes of surgical removal. Samples were either embedded in Tissue-Tek Optimal Cutting Temperature Compound (Sakura Finetek U.S.A., Inc.) and rapidly frozen and stored at −70°C (frozen sections) or were fixed in formaldehyde and processed for paraffin embedding.

The alkaline phosphatase immunohistology technique was described previously ([75]). Two anti-FcRn antibodies were used: 1) Anti-FcRn antibody purified from rabbit serum raised against α2 (88–177aa) and α3 (1782-247aa) domains of human FcRn (provided by Neil Simister, Brandeis University) for use on frozen sections (**Figure 5**), and 2) rabbit anti-FcRn antibody obtained from Novus Biologicals for use on paraffin sections following citrate buffer (pH 6.0) antigen retrieval (**Figure S7**). Sections were blocked with serum-free protein solution, and optimally diluted primary FcRn antibodies or rabbit IgG (negative control) were added and incubated for 1–2 hours at RT. Binding of antibodies to FcRn in tissues was visualized using an alkaline phosphatase detection system that stains positive cells bright red. Sections were counterstained with hematoxylin and cover-slipped using aqueous mounting medium.

### Statistics

Differences in amounts of transcytosed or infectious virus between conditions were compared using Kruskal-Wallis or repeated-measures ANCOVA. For repeated-measures ANCOVA, the percentage of transcytosed or infectious virus was logit-transformed and normality evaluated using the Shapiro–Wilk test. Correlations between continuous variables were evaluated by Spearman's *rho*. Two-tailed *p*-values are reported.

## Supporting Information

Figure S1 Transcytosis of HIV-1 at pH 6.0 over time. HIV-1_JRFL_ with HIVIG, IVIG or no antibody was applied to the apical surface of HEC-1A cells at pH 6.0. At the indicated times, subnatant fluid (pH 7.4) was collected, and transcytosis was quantified by RT-PCR.
(PPTX)

Figure S2 Antibody and low pH-mediate enhanced transcytosis of HIV-1 across T84 colonic carcinoma cells. HIV-1_US657_ and the indicated concentrations of HIV-specific IgG (HIVIG) or control IgG (IVIG) were applied to the apical surface of T84 colonic carcinoma cells at pH 6.0 or 7.4. After 12 hours, virus in the subnatant fluid was quantified by RT-PCR and expressed as a percentage of the inoculum added to the apical surface. Data represent the mean+SE of two independent experiments. Similar results were obtained in an additional experiment using another R5 strain of HIV-1 (HIV-1_US712_).
(PPTX)

Figure S3 Enhanced transcytosis is due to FcRn. (**A**) Knockdown of HEC-1A cells was accomplished using lentivirus particles expressing small hairpin RNA and verified by flow cytometry (left panel) and Western blot (right panel) using rabbit polyclonal anti-FcRn antibodies; WT = wild-type HEC-1A cells and KD = FcRn-knockdown HEC-1A cells. GAPDH was used as a protein-loading control. (**B**) Binding of wild-type mAb b12 and Fc mutants of b12 (M428L and I253A) to HIV-1_JRFL_ gp120 by ELISA is equivalent. (c) Neutralization of HIV-1_JRFL_ by wild-type b12 and its Fc mutants are similar. IC_50_ values for wild-type b12, M428L, and I253A are 0.03, 0.04, and 0.02 µg/ml, respectively. (**D–F**) Enhanced transcytosis of HIV-1 is blocked by bafilomycin A1 and by an anti-FcRn antibody. HIVIG and HIV_US657_ (**D**) or HIVIG or mAb b12 and HIV_US712_ (**E**, **F**) were added to the apical surface of HEC-1A cells at pH 6.0 in the presence or absence of 0.1 µM bafilomycin A1 (**D**–**F**) or in the presence of rabbit polyclonal anti-FcRn IgG (50 µg/ml) or normal rabbit polyclonal IgG (“Neg antibody”) (**E**, **F**). Data represent mean + SE of two independent experiments (**D**) or single experiments (**E**, **F**). (**G**) Non-HIV-specific mAb (Den3) inhibits VRC01-mediated enhancement of transcytosis of HIV-1 at pH 6.0. Immune complexes, made with 25 µg/ml of VRC01 and HIV-1_JRFL_, were added to the apical surface of HEC-1A cells in the presence of indicated concentrations of Den3 at pH 6.0. Subnatant fluid (pH 7.4) was collected 12 hours later, and virus was quantified by RT-PCR. Numbers above the bars indicate the % inhibition of transcytosis. Data represent mean + SE of two independent experiments. (**H**) Maximum transcytosis in the presence of HIV-1-specific antibody occurs at pH 5.5–6.0. HIV-1_JRFL_ was incubated with HIVIG (50 µg/ml) at indicated pH values and added to the apical surface of HEC-1A cells. Transcytosis was quantified by RT-PCR in subnatant fluid collected 6- and 12-hours later. Note that IVIG and no antibody controls, used at pH 6.0 only, gave similarly low levels of transcytosis compared to HIVIG at pH 7.4 (not shown).
(PPTX)

Figure S4 Transcytosis of IgG alone is influenced by pH at the apical cell surface. 50 µg/ml of mAbs Den3 or VRC01 were added to the apical side of HEC-1A cells. Twelve hours later, IgG was quantified in the subnatant fluid by ELISA.
(PPTX)

Figure S5 Transcytosis of HIV-1_JRFL_ correlates directly with mAb binding and inversely with neutralizing activity. (**A**) mAb binding by ELISA to HIV-1_JRFL_ directly coated on plates. (**B**) Correlation between mAb binding and transcytosis at 50 µg/ml of mAb (Spearman *rho* = 0.75; *p* = 0.052). (**C**) Correlation between IC_50_ (**see** **Figure 3E**) and transcytosis at 50 µg/ml of mAb (Spearman *rho* = −0.71; *p* = 0.050).
(PPTX)

Figure S6 Anti-monomeric Env binding activity of CVL and seminal fluid IgG as a percentage of total IgG. The dotted line represents a negative cutoff based on the mean+2 SD of IgG from cervicovaginal and seminal fluid of the HIV-uninfected subjects. Shown is the mean+SE of two independent assays, each done in duplicate.
(PPTX)

Figure S7 Additional examples of FcRn expression in the columnar epithelia of human penile urethra and endocervix. (**A**) Penile, (**B**) endocervical or (**C**) vaginal tissue was stained with affinity-purified anti-human-FcRn rabbit IgG or with normal rabbit IgG (**D, E**). Staining was performed on frozen tissue sections.
(PPTX)
