# Correction: The Neonatal Fc Receptor (FcRn) Enhances Human Immunodeficiency Virus Type 1 (HIV-1) Transcytosis across Epithelial Cells

**DOI:** 10.1371/annotation/31430955-703b-484a-96eb-e180f917d683

**Published:** 2013-11-27

**Authors:** Sandeep Gupta, Johannes S. Gach, Juan C. Becerra, Tran B. Phan, Jeffrey Pudney, Zina Moldoveanu, Sarah B. Joseph, Gary Landucci, Medalyn Jude Supnet, Li-Hua Ping, Davide Corti, Brian Moldt, Zdenek Hel, Antonio Lanzavecchia, Ruth M. Ruprecht, Dennis R. Burton, Jiri Mestecky, Deborah J. Anderson, Donald N. Forthal

The following sentences in the main text use an incorrect unit of measure:

"Electrical resistance across the membrane, which ranged from 400-450 mOhms/cm2 at the start of the transcytosis assay, confirmed monolayer integrity. Resistance was re-measured after the transcytosis assay in more than 50% of wells and ranged from 450-480 mOhms/cm2."

The correct sentences are:

"Electrical resistance across the membrane, which ranged from 400-450 Ohms at the start of the transcytosis assay, confirmed monolayer integrity. Resistance was re-measured after the transcytosis assay in more than 50% of wells and ranged from 450-480 Ohms."

Figure S3 contains a duplicate of the file contained in Figure S2. Please use the following link to download the correct version of Figure S3: 

. 

