# Supplementary material for: Correction: The Neonatal Fc Receptor (FcRn) Enhances Human Immunodeficiency Virus Type 1 (HIV-1) Transcytosis across Epithelial Cells
Source: PLoS Pathog. 2013 Nov 27;9(11):10.1371/annotation/31430955-703b-484a-96eb-e180f917d683. doi: 10.1371/annotation/31430955-703b-484a-96eb-e180f917d683 (PMC3842409; doi:10.1371/annotation/31430955-703b-484a-96eb-e180f917d683)
Supplement: Supplementary file 1 [file ppat.31430955-703b-484a-96eb-e180f917d683.s001.pptx]

## Slide 1
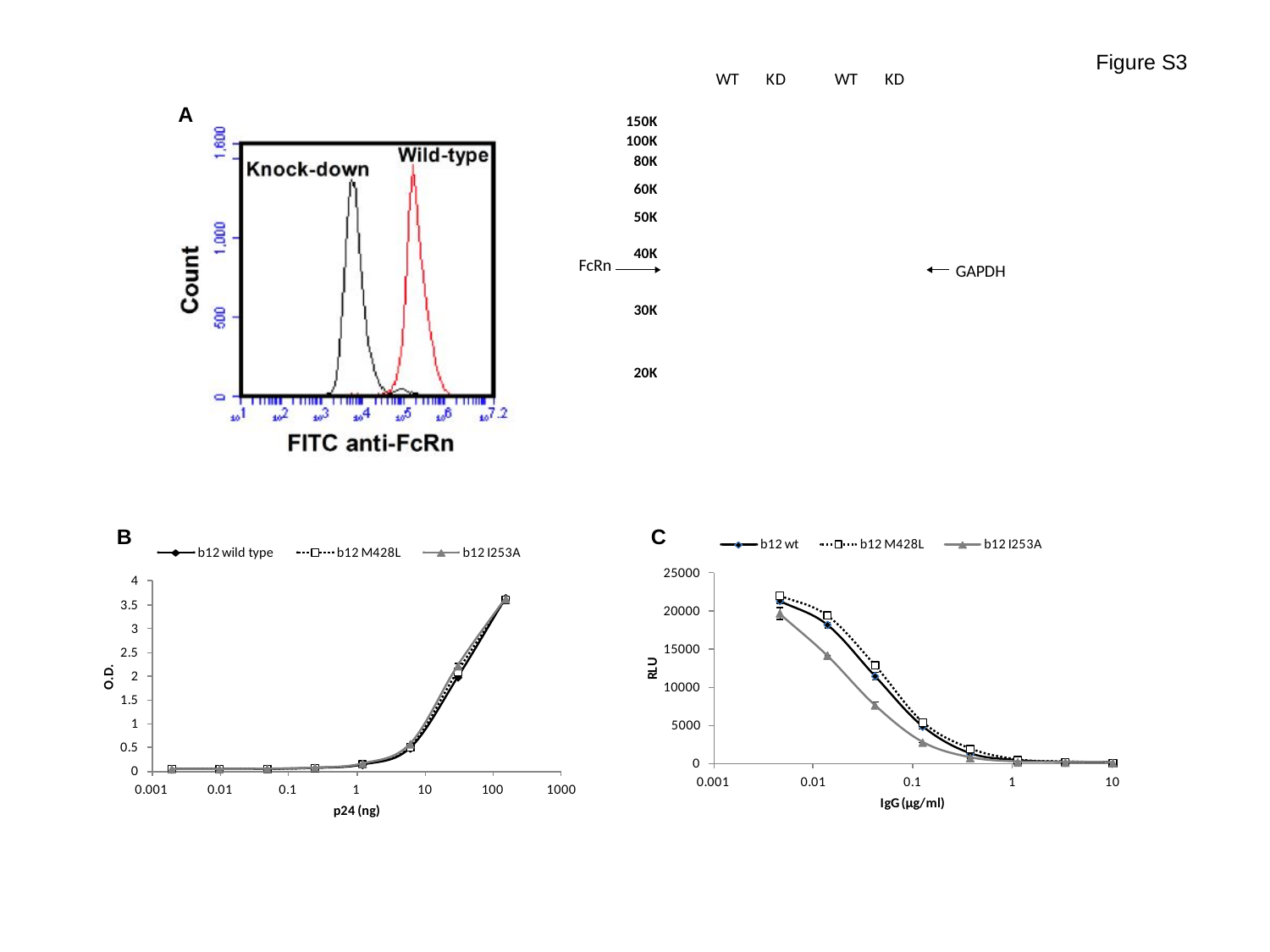

Figure S3
A
C
B

## Slide 2
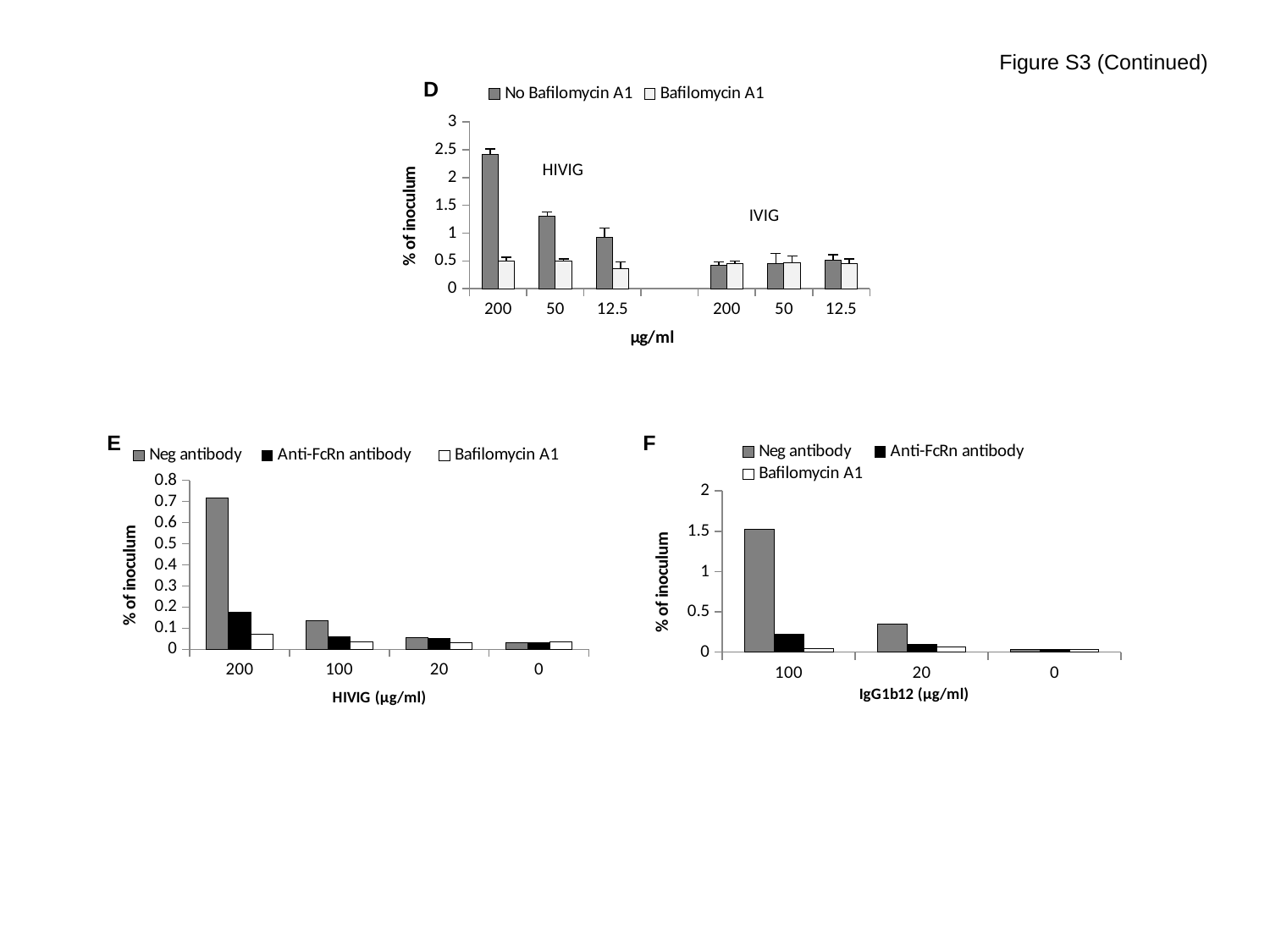

Figure S3 (Continued)
D
### Chart
| Category | No Bafilomycin A1 | Bafilomycin A1 |
|---|---|---|
| 200 | 2.409389796278811 | 0.49005698115899565 |
| 50 | 1.312046753591036 | 0.48990211521148236 |
| 12.5 | 0.9270944788778548 | 0.36646945818455307 |
| | None | None |
| 200 | 0.41597759641948623 | 0.45023784096241826 |
| 50 | 0.4533807854863097 | 0.46148193148786054 |
| 12.5 | 0.5077370031106285 | 0.45849040210739644 |HIVIG
IVIG
E
F
### Chart
| Category | Neg antibody | Anti-FcRn antibody | Bafilomycin A1 |
|---|---|---|---|
| 100 | 1.5259250342835682 | 0.2213046468120009 | 0.047992470962850454 |
| 20 | 0.35319002226902985 | 0.10012932423884843 | 0.06132140682501942 |
| 0 | 0.031896423345817035 | 0.03270657479515186 | 0.03418964822575899 |
### Chart
| Category | Neg antibody | Anti-FcRn antibody | Bafilomycin A1 |
|---|---|---|---|
| 200 | 0.7180153487781669 | 0.17688786772356238 | 0.07206746934984777 |
| 100 | 0.1348390826240964 | 0.060244129311898674 | 0.036465981310490905 |
| 20 | 0.053930919060277575 | 0.051586756906407884 | 0.031064797056060252 |
| 0 | 0.031896423345817035 | 0.03270657479515186 | 0.03418964822575899 |

## Slide 3
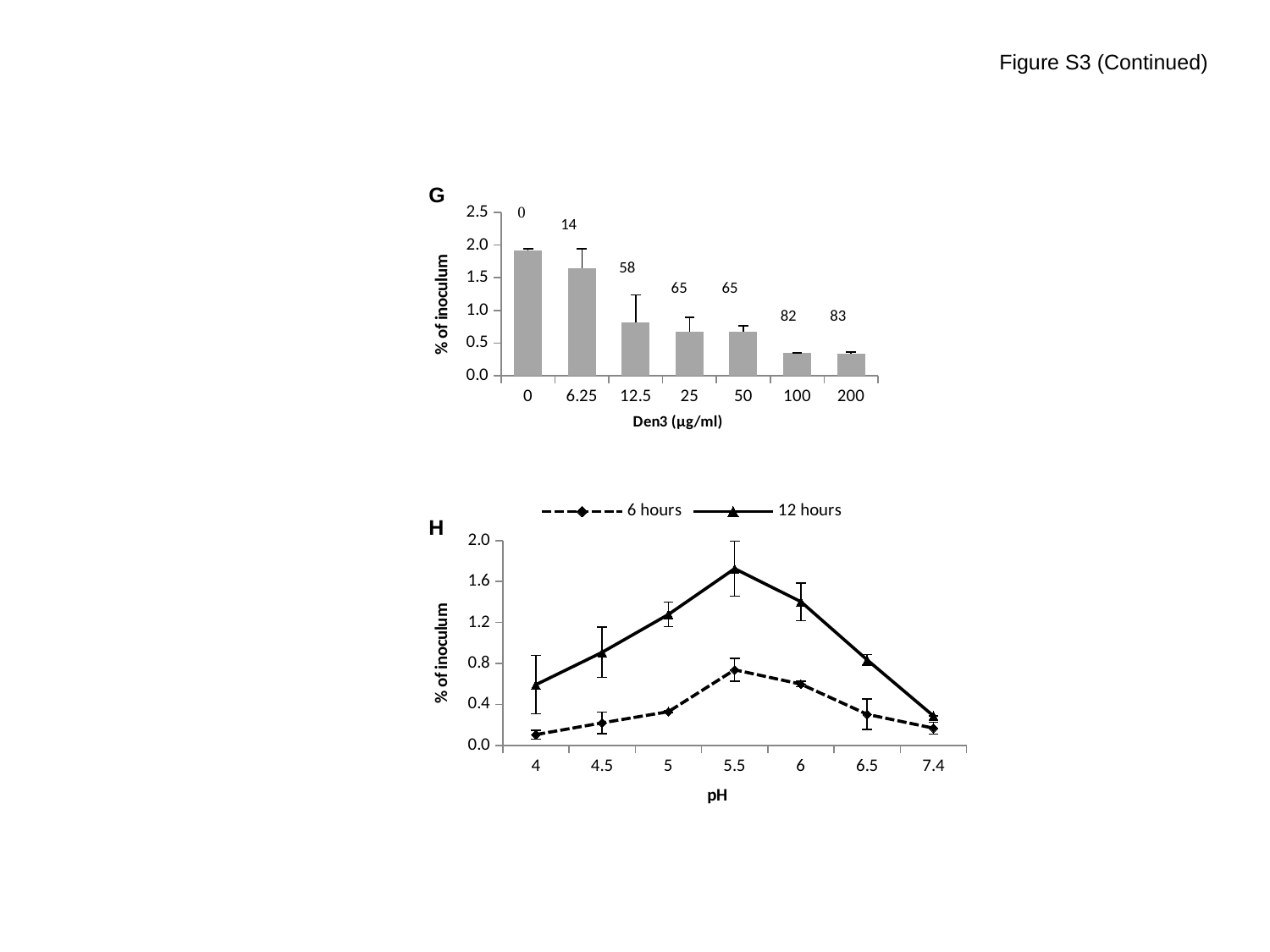

Figure S3 (Continued)
G
0
14
58
65
65
82
83
### Chart
| Category | |
|---|---|
| 0 | 1.9178934924943754 |
| 6.25 | 1.651780039199581 |
| 12.5 | 0.8143623380600356 |
| 25 | 0.6741204355240082 |
| 50 | 0.6753391946607686 |
| 100 | 0.3478266765850209 |
| 200 | 0.3353525936525304 |
### Chart
| Category | 6 hours | 12 hours |
|---|---|---|
| 4 | 0.10565074268258379 | 0.5927122718297461 |
| 4.5 | 0.21999111650368294 | 0.9093084239624799 |
| 5 | 0.32953666043825786 | 1.2788119398532054 |
| 5.5 | 0.7379748694713812 | 1.7258174671180084 |
| 6 | 0.5982274951266875 | 1.4031945719301437 |
| 6.5 | 0.30355907507977686 | 0.8345587459538475 |
| 7.4 | 0.1687130961236154 | 0.2880401546900701 |H
